# AI4Hope: Advanced Care Planning and Virtual Reality for People with Dementia

**DOI:** 10.1002/alz70858_107256

**Published:** 2025-12-26

**Authors:** Martin Rakusa, Aljaz Hölbl, Sofia Rakusa

**Affiliations:** ^1^ University Medical Centre Maribor, Maribor, Slovenia

## Abstract

**Background:**

AI4Hope is a research project integrating digital health interventions, including virtual reality (VR) and biosignal‐based care planning, to enhance dementia management and improve the quality of life of people with dementia (PWD). Successful implementation of these technologies requires understanding healthcare professionals’ perspectives and readiness for adoption. This study evaluates the feasibility of advanced care planning and VR as supportive tools in dementia care.

**Method:**

Nurses working in neurological and psychiatric wards, all actively caring for patients with cognitive impairment and dementia, were included. A nine‐question survey assessed current care resources, the role of VR in cognitive engagement and stress reduction, and ethical considerations such as data privacy using a Likert scale. Responses were analyzed using frequency analysis.

**Result:**

Fifty nurses participated, equally divided between those with high school and faculty‐level education, with 45% aged over 45 years. Three‐quarters of respondents reported using smartwatches, health‐tracking wristbands, or VR tools. Most (94%) believed additional technological solutions were needed, and all agreed that tools should be adapted to different dementia stages. Most (82%) found wearable devices helpful in monitoring patients, and nearly all (94%) supported their use for tracking mood, activity, and distress to improve patient care. VR was perceived as beneficial for relaxation and stress reduction (71%), with preferred VR environments including natural landscapes (65%), cultural experiences (47%), and social simulations (35%). A majority (82%) believed digital tools enhance care planning, while 88% emphasized the importance of accessibility. All respondents (100%) agreed these tools should facilitate communication among healthcare providers and caregivers. Privacy protection was considered essential (88%); 41% expressed ethical concerns. All respondents (100%) expressed the need for training before implementing digital solutions in dementia care.

**Conclusion:**

This study demonstrates nurses’ strong acceptance of digital health interventions and their potential benefits in dementia care. However, effective implementation requires targeted training to ensure that healthcare professionals can fully utilize these technologies.

Co‐funded by the European Union Horizon Europe (AI4HOPE, 101136769), UK Research and Innovation (Grant No. 101136769).

